# Biomarkers for cancer-associated fibroblasts

**DOI:** 10.1186/s40364-020-00245-w

**Published:** 2020-11-11

**Authors:** Chencheng Han, Tongyan Liu, Rong Yin

**Affiliations:** 1 Department of Thoracic Surgery, The Affiliated Cancer Hospital of Nanjing Medical University & Jiangsu Cancer Hospital & Jiangsu Institute of Cancer Research, Jiangsu Key Laboratory of Molecular and Translational Cancer Research, Nanjing, China; 2 Department of Science and technology, The Affiliated Cancer Hospital of Nanjing Medical University & Jiangsu Cancer Hospital & Jiangsu Institute of Cancer Research, Jiangsu Key Laboratory of Molecular and Translational Cancer Research, Nanjing, China; 3 Biobank of Lung Cancer, Jiangsu Biobank of Clinical Resources, Nanjing, China; 4 Collaborative Innovation Center for Cancer Personalized Medicine, Nanjing Medical University, Nanjing, China

**Keywords:** Biomarker, Cancer-associated fibroblasts, Heterogeneity

## Abstract

Cancer-associated fibroblasts (CAFs) are the key component of tumor stromal. High heterogeneity of CAFs reflects in their origin, phenotype and function. Biological function which can be suggested by biomarkers of distinct CAF subgroups may be different, even opposite, just like water and fire. Identifying CAF subpopulations expressing different biomarkers and reconciling the relationship of the “water and fire” among distinct CAF subsets may be a breakthrough in tumor therapy. Herein, we briefly summarize the biomarkers commonly used or newly identified for distinct CAFs in terms of their features and potential clinical benefits.

As the most abundant and main component in the tumor microenvironment (TME), cancer-associated fibroblasts (CAFs) are generally considered as all the fibroblasts found within and surrounding tumor tissues, which are activated from normal resident tissue fibroblasts or transdifferentiated from non-fibroblastic lineage such as epithelial cells and adipocytes due to the stimulation of TME [1, 2]. CAFs were thought to be tumor-promoting by building up and remodeling extracellular matrix (ECM). However, latest study revealed the extensive inter- and intra-organ heterogeneity of fibroblasts in the physiological context [3], and several preclinical studies attempted to target CAFs directly in mouse models also failed [4, 5]. These evidences suggest an obvious heterogeneity of CAFs which may harbor both tumor-promoting and anti-tumor properties.

Traditional CAF biomarkers such as α-smooth muscle actin (α-SMA), fibroblast activation protein (FAP), S100A4, platelet-derived growth factor receptors (PDGFRα/β) or vimentin have been well-studied despite none of them are specific to CAFs (Table 1) [6]. Moreover, increasing CAF subsets with distinct biomarkers expression and different cellular functions have been identified recently. We here briefly outline the biomarkers for identifying CAF heterogeneity and potential therapeutic targets.

**Table 1:** List of commonly used biomarkers for CAFs

Marker	Cell origin	Biological effects	Effects on tumors	Clinical application
Neutral biomarkers with dual functions				
α-SMA	Normal fibroblasts, quiescent stellate cells, smooth muscle cells	Cell contractility, structure and integrity, desmoplasia	Tumor proliferation, immunosuppressive; protection mechanism, impeding drug delivery	Preclinical trials by targeting α-SMA directly failed; prognostic indicator
S100A4	Normal fibroblasts, epithelial cells, endothelial cells	Cell motility, tissue fibrosis	Promoting metastasis, immune evasion; immune surveillance and response	Unknown
Accomplices: pro-tumorigenesis biomarkers				
FAP	Normal fibroblasts, quiescent stellate cells, CD45^+^ immune cells	ECM remodeling, fibrogenesis, serine protease activity	Tumor progression and metastasis, shaping the immunosuppressive TME	Preclinical trials (antibody, inhibitor, DNA vaccination, oncolytic adenovirus, CAR-T); phase II clinical trials (PT-100, sibrotuzumab) failed; prognostic indicator
PDGFRα/β	Normal fibroblasts, vascular smooth muscle cells, pericytes	Receptor tyrosine kinase activity	Immunomodulation, M2 polarization, angiogenesis	Dasatinib normalizes CAFs; targeting Saa3 in PDGFRα^+^ CAFs; prognostic indicator
PDPN	Endothelial cells	Cell motility and adhesion	Immunosuppressive	Unknown
CD70	T and B lymphocytes, mature dendritic cells	T cell function regulation	Immunosuppressive, tumor cell migration, T cell exhaustion	Prognostic indicator
Vimentin	Epithelial cells, endothelial cells	Cell motility, structure and integrity	Tumor invasion	Unknown
GPR77	Polymorphonuclear neutrophils	Complement activation, pro-inflammatory signaling	Sustaining cancer stemness, cancer formation, chemoresistance	Neutralizing anti-GPR77 antibody abolishes tumor formation in a PDX model
CD10	BMSCs, pre-B lymphocytes	Metalloendoprotease	Sustaining cancer stemness, cancer formation, chemoresistance	Unknown
CD74	Normal fibroblasts, monocytes/macrophages, epithelial cells	MHC II chaperone, protein trafficking	Immunomodulation	Unknown
Defenders: tumor-suppressive biomarkers				
CD146	Endothelial cells	Cell adhesion	Maintaining ER expression, sensitive to tamoxifen	Prognostic indicator; Considered as a drug?
CAV1	Normal fibroblasts, endothelial cells, adipocytes	Structure component, cell signaling and transport	CAV1^low^ associated with poor prognosis	Prognostic indicator
Saa3^−^	Smooth muscle cells, adipocytes	Collagenase production	PDGFRα^+^Saa3^−^ CAF impairing tumor proliferation	Targeting Saa3 in PDGFRα^**+**^ CAF; prognostic indicator

## CAFs isolation and characterization

CAFs can be easily digested and cultured on plastic flasks, whereas other types of cells not, which is the basis of CAFs isolation [7]. Briefly, obtained tumor tissues are minced into small pieces about 1 mm and then digested at 37 °C with shaking. Usage of gentle tissue dissociators may improve separation efficiency. For digestion, diverse collagenases, trypsin, hyaluronidase and dispase can be used alone or combined. The cells acquired are filtered through cell strainers and then planted in culture plates. Breast tumor tissues are usually incubated at room temperature for 5 min without shocking after digestion. Red blood cell lysis buffer is optional.

The primary CAFs should be negative for epithelial (EpCAM), endothelial (CD31) and leukocyte (CD45) with an elongated spindle-like morphology [1]. In practice, traditional CAF biomarkers are typically combined with lineage exclusion to identify CAFs. Notably, the passage number of cultured CAFs between 1 to 6 is suitable for experiments.

## Neutral biomarkers with dual functions

### α-SMA: a snapshot of CAF heterogeneity

As the marker of activated fibroblasts, α-SMA, a cytoskeletal protein associated with TGF-β production and highly contractile phenotype, is the first identified and most frequent CAF biomarker [8, 9]. Studies have verified that α-SMA^+^ CAFs could promote tumor progression, confer therapeutic resistance and mediate immunosuppressive TME in multiple ways, such as paracrine and ECM remodeling [2, 10, 11].

However, depleting α-SMA^+^ CAFs directly has been failed to treat pancreatic ductal adenocarcinoma (PDAC) in murine models, probably due to the immune surveillance suppression [4]. Patients with high desmoplasia defined by expression of collagen I and CAF markers including α-SMA have better prognosis in PDAC, breast and lung cancer patients [12, 13]. These results show that the fibrotic response of α-SMA^+^ CAFs may be a host protection mechanism against tumor progression, which needs further investigation.

**Fig. 1:**
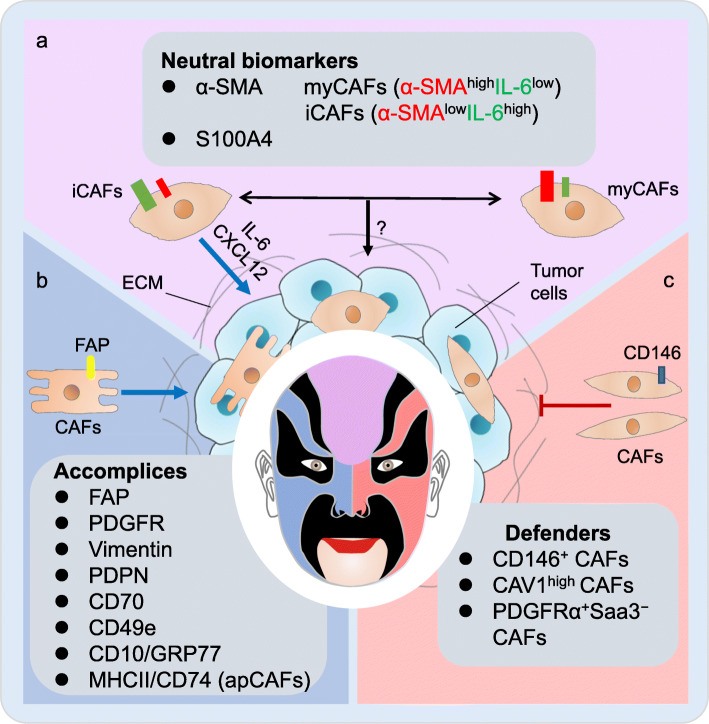
The “double face” of cancer-associated fibroblasts (CAFs). CAF subsets identified by different biomarkers play distinct roles in tumor microenvironment. **a**, several studies have shown that CAFs expressing α-SMA or S100A4, which are considered tumorigenic previously, are also potentially anti-tumor. Identification of myCAFs and iCAFs among α-SMA^+^ CAFs may represent an aspect of CAF heterogeneity, which is believed that two subsets can convert into each other. **b**, most biomarkers represent tumor-promoting CAFs including traditional CAF biomarkers (FAP, PDGFR, Vimentin, PDPN and CD70) as well as some newly identified markers (CD49e, CD10/GRP77 and MHCII/CD74). **c**, CD146^+^ CAFs, CAV1^high^ CAFs and PDGFRα^+^Saa3^−^ have been identified as tumor-suppressive CAF subsets

The contradictory conclusions above indicate the α-SMA^+^ CAF heterogeneity which has been confirmed in a recent study classifying different subtypes of α-SMA^+^ CAFs. Typically, inflammatory CAFs (iCAFs) being α-SMA^low^IL-6^high^ are identified in PDAC and located more distantly from neoplastic cells than α-SMA^high^IL-6^low^ myofibroblastic CAFs (myCAFs) (Fig. 1a). ICAFs appear to be more tumor-promoting than myCAFs by producing chemokines and cytokines [14] and indicate a higher malignancy in pancreatic tumorigenesis [15]. On the other hand, myCAFs may deposit ECM extensively to impede drug delivery despite less cancer-stimulating [16]. Considering the biological effects of myCAFs and iCAFs, the composition of myCAFs and iCAFs in the TME may have different implications for treatment. High iCAF content may indicate a higher degree of malignancy, while high myCAF content may indicate a poor response to treatment.

Accordingly, combination of reducing the upstream formation of iCAFs with inhibiting the downstream desmoplasia derived from myCAFs may design the treatment strategy. Furthermore, IL-1/JAK/STAT signaling cascades and TGF-β have been found involved in the formation and mutual transformation of these two CAF subtypes. JAK inhibitors can suppress tumor growth as well as shift iCAFs to myCAFs while TGFBR inhibition could partially attenuate the function of myCAFs without influencing tumor growth [16]. Patients might benefit from combination therapy of these two drugs. Altogether, classification of α-SMA^+^ CAFs deepens our understanding of CAF heterogeneity as well as provides new ideas for CAF targeted therapy which remains further study.

### Bidirectional roles of S100A4

S100A4, also called fibroblast-specific protein-1, is usually expressed on CAFs transited from epithelial or endothelial cells [17, 18]. However, the biological effects of S100A4^+^ CAFs are controversial (Fig. 1a). S100A4^+^ CAFs promote tumor metastasis through secretion of VEGF- A and Tenascin-C [19]. Chemokine ligand 2 (CCL2) derived from S100A4^+^ CAFs contributes to immune evasion by maintaining macrophage infiltration [20]. On the other hand, fibroblasts expressing S100A4 can product collagen and encapsulate carcinogens to enhance immune surveillance ability [21]. α-SMA^+^S100A4^+^ CAFs can activate tumor immune response by promoting CD8^+^ T cell activation through fusion with dendritic cells [20]. These findings confirmed the CAF heterogeneity, and S100A4^+^ CAFs remain further characterization.

## Accomplices: pro-tumorigenesis biomarkers

### FAP: a promising therapeutic target

FAP is another wide-spread biomarker for CAFs, as well as a serine protease participating in ECM remodeling and fibrogenesis, thereby accelerating tumor progression [22]. FAP^+^ CAFs can shape the immunosuppressive TME by secreting distinct chemokines and cytokines [2]. A recent study in ovarian cancer found that FAP^high^ CAFs are correlated with poor patient outcomes [23].

So far, FAP might be one of the most promising therapeutic targets of CAFs. In distinct murine tumor models, multiple strategies targeting FAP exhibited therapeutic effects, including genetic deletion [24], pharmacological inhibition (PT630, PT-100) [25, 26], a novel monoclonal antibody (mAb) FAP5-DM1 [27], conditional ablation of FAP^+^ CAFs using diphtheria toxin [28] or αFAP-PE38 [29], and even novel FAP-targeting immunotherapies such as DNA vaccination [30], chimeric antigen receptor (CAR) T cells [31, 32] or oncolytic adenovirus [33, 34].

However, sibrotuzumab, a FAP-specific antibody, which has been found clinically safe and effective in a phase I trial of advanced cancers, showed no beneficial effect in a phase II trial of metastatic colorectal cancer [35–37]. Another phase II trial utilizing PT-100 in metastatic colorectal cancer also failed [38]. It is noted that the patients involved in both of these phase II trials were heavily pre-treated and represented a refractory patient population, which may account for the failure. FAP may probably contribute more in the earlier-stage tumors rather than late-stage metastasis [39]. Nevertheless, FAP is still the most promising CAF therapeutic target and more exploration is needed.

### Other traditional biomarkers: PDGFR, Vimentin, PDPN and CD70

PDGFRα/β are both upregulated in multiple tumors [40], and PDGFRβ is correlated with poor survival in breast cancer [41]. PDGFRα/β^+^ CAFs participate in immunomodulation by inducing macrophage migration and M2 polarization [2]. Blocking PDGFR signaling can suppress angiogenesis and tumor growth in human cervical cancer [42]. Furthermore, a PDGFR inhibitor, Dasatinib can partially reverse the pro-tumorigenic CAFs of lung adenocarcinoma (LUAD) to a quiescent state [43], which might be a potential treatment strategy for LUAD.

Vimentin is a biomarker for epithelial-to-mesenchymal transition (EMT) maintaining structure and motility during cell migration [44], involved in CAF motility to lead cell invasion in LUAD [45]. Podoplanin (PDPN)^+^ CAFs in LUAD have been reported as the inducer of immunosuppressive microenvironment [46]. Moreover, CAFs expressing CD70 enrich regulatory T cells to invasive colorectal cancer and CD70 expression is negatively correlated with survival of patients with colorectal cancer (Fig. 1b) [47]. PDPN seems to be a potential therapeutic target while CD70 and vimentin might be more suitable for prognostic indicators and more evidences are warranted.

## Defenders: tumor-suppressive biomarkers

The potential anti-tumor function of CAFs exhibits when we attempt to inhibit hedgehog pathway which has been proved to activate fibroblasts [48–50], indicating the existence of tumor-suppressive CAF subsets. Studies on CAF heterogeneity has identified several potential anti-tumor CAF subpopulations and biomarkers (Fig. 1c).

CD146 expression is found to distinguish at least two CAF subpopulations in ER^+^ breast cancer, among which CD146^+^ CAFs could promote tamoxifen sensitivity by continuously expressing ER, whereas CD146^−^ CAFs opposite [51]. Another stroma-derived gene expression signature of breast tumor shows that CAV1^low^ CAFs are correlated with poor prognosis [52]. Serum amyloid A3 (Saa3) is also identified as new biomarker of mouse PDGFRα^+^ CAFs in PDAC, as PDGFRα^+^Saa3^+^ CAFs could facilitate tumor growth while PDGFRα^+^Saa3^−^ CAFs impairing tumor proliferation [53]. In summary, increasing proportion of CD146^+^ CAFs in ER^+^ breast cancer and targeting Saa3 specifically in PDAC might be promising strategies to revert the TME to an anti-tumor environment. So far it might be too immaturely to use the defensive CAFs as a cellular therapeutic strategy, however at least these biomarkers might be benefit for prognostic diagnosis.

## Newly identified CAF subpopulations and biomarkers

Recent advances using single-cell RNA sequencing (scRNA-seq) provide us technical advantages to better understand CAF heterogeneity and identify novel biomarkers. For example, CD49e has been identified as a new cell surface pan-CAF biomarker in ovarian cancer recently, just like α-SMA [23]. Herein, we list the CAF subpopulations and biomarkers newly identified in distinct tumor tissues (Table 2).

**Table 2:** List of CAF subpopulations and biomarkers newly identified

CAF subsets	Biomarkers	Tumor tissues	Signatures/functions	Refs
pan-CAF	CD49e	Ovarian cancer		[23]
myCAF	α-SMA^high^IL-6^low^; RGS5	PDAC; TNBC; bladder cancer	Myofibroblast-like; matrix deposition;	[14, 56, 58]
iCAF	α-SMA^low^IL-6^high^; PDGFR; CXCL12; Ly6c1^high^; FBLN1	PDAC; TNBC; bladder cancer; ICC	Inflammatory infiltration; chemokines and cytokines secretion; tumor-promoting	[14, 56–59]
apCAF	MHC-II gene; CD74	PDAC; TNBC; ICC	Antigen presenting; immunomodulation	[57–59]
vCAF	MCAM; IL-6	ICC	Response to hypoxia; mesenchymal cell proliferation	[59]
mCAF	POSTN; COL5A1		ECM; collagen fibril organization	
EMT-like CAF	KRT19		Epithelium-like	
vCAF	Nidogen-2	Breast cancer	Vascular development; angiogenesis	[60]
mCAF	Fibulin-1; PDGFRα		ECM and EMT	
developmental CAF	SCRG1		Differentiation of cells; development and morphogenesis of tissues	
CAF-A	MMP2; DCN; COL1A2	Colorectal cancer	ECM remodeling	[61]
CAF-B	α-SMA; TAGCN; PDGFA		Myofibroblast-like	
CAF-cluster2	CDK1	TNBC	Cell cycling	[58]
CAF-cluster3	CD53		Structural integrity and function of muscle	
CAF-cluster4	CRABP1		Basement membrane protease associated	
Immunomodulatory CAF	IL-6; IL-10; C1QA/B/C; CFB; CXCL1/2/10/12	HGSOC	Immunomodulation	[62]
CAF-S1/S4	CD29; FAP; α-SMA; FSP1; PDGFRβ; CAV1	Breast cancer; ovarian cancer	Immunomodulation; myCAFs and iCAFs	[63–66]
CAF-S2/S3			Not activated	
CD10^+^GPR77^+^ CAFs	CD10; GPR77	Breast and lung cancer	Promoting cancer formation and chemoresistance	[54]

GPR77 and CD10 are potential targeted biomarkers as the infiltration of CD10^+^GPR77^+^ CAFs indicates chemotherapy resistance and poor survival, especially in the ER^−^HER2^−^ subtype and high-grade breast tumors. Blocking GPR77 substantially can suppress tumorigenesis along with enhancing chemosensitivity in a patient-derived xenograft model [54].

The existence of iCAFs and myCAFs has been verified in triple-negative breast cancer (TNBC) and bladder urothelial carcinoma by scRNA-seq, despite the biomarkers identified are different. For specific, CX-chemokine ligand 12 (CXCL12) is the biomarker of TNBC iCAFs [55]. RGS5 and PDGFR are the biomarkers for myCAFs and iCAFs in bladder tumor, respectively [56].

Major histocompatibility complex (MHC) class II family genes and CD74 have been identified as biomarkers of another PDAC CAF subpopulation termed as antigen presenting CAFs (apCAFs) besides myCAFs and iCAFs. ApCAFs process an immunomodulatory role by interacting with CD4^+^ T cells [57]. All of these three CAF subpopulations with another three are verified in TNBC. It is noted that apCAFs are also found in normal tissues, indicating that the phenotype is not TME-induced. Furthermore, PDGFRα is found highly expressed in iCAFs while PDGFRβ is found highly expressed in myCAFs [58].

A recent scRNA-seq study conducted in human intrahepatic cholangiocarcinoma (ICC) found five CAF subpopulations: vascular CAFs (vCAFs) defined by MCAM expressing high level of IL-6, matrix CAFs (mCAFs) defined by POSTN, iCAFs defined by FBLN1, apCAFs defined by CD74 and EMT-like CAFs defined by KRT19 [59]. Another study identified three CAF subpopulations with distinct biomarkers in breast cancer: vCAFs with marker Nidogen-2, mCAFs with marker PDGFRα and developmental CAFs with marker SCRG-1 [60]. Moreover, two CAF subpopulations are detected in colorectal tumors: CAF-A expressing ECM remodeling genes such as MMP2, DCN, COL1A2 and CAF-B expressing markers of myofibroblasts such as α-SMA, TAGLN, PDGFA [61]. PDPN, DCN and THY1 are another group of biomarkers classifying four CAF subpopulations in high-grade serous ovarian cancer (HGSOC). Immunomodulatory CAFs expressing highly IL-6/CXCL12 identified could activate JAK/STAT signaling in tumor cells [62], just like iCAFs.

Mechta-Grigoriou et al. characterized four CAF subsets in breast, ovarian cancers and metastatic lymph node of breast cancer with distinct properties by analyzing six fibroblast biomarkers (FAP, α-SMA, β1/CD29, S100A4, PDGFRβ, and CAV1). The identified CAF-S1 subset can promote an immunosuppressive environment and stimulate migration of cancer cells [63–65]. Further investigation classified the CAF-S1 subset into 8 different clusters by scRNA-seq, among which three clusters belong to the iCAFs while another 5 clusters belong to the myCAFs [66].

As we summarized above, increasing CAF subsets with distinct biomarkers have been identified to coexist in tumor tissues and play different biological functions. However, due to the tissue heterogeneity, distinct classification criteria, biomarkers and nomenclature selected by different laboratories, the identification of CAF subsets is somewhat messy and intersecting at present. Among the CAF subsets identified, myCAFs and iCAFs both seem prevalent across-organ despite the biomarkers identified are different, probably due to the tissue heterogeneity. Furthermore, iCAFs have been widely proved to promote tumor progression by secreting chemokines and cytokines such as IL-6 and CXCL12. On the other hand, several CAF subgroups identified from different tissues have been named the same by different studies, such as vCAFs and mCAFs. However, given their different biological functions and biomarkers, whether they are the same subgroup is worth further discussion. We believe that with the further research on the CAF heterogeneity, there will be a unified standard for the selection of CAF biomarkers and nomenclature of CAF subpopulations.

With the further development of the technology, we believe that our understanding of the CAF subsets and biomarkers will be more profound. CAF biomarkers can be utilized as both prognostic indicators and therapeutic targets for clinical benefits. Hopefully, we can reverse the accomplice CAFs to defender ones by targeting appropriate molecules.

## Data Availability

Not applicable.

## Abbreviations

CAF: Cancer-associated fibroblast
TME: Tumor microenvironment
ECM: Extracellular matrix
α-SMA: Αlpha-smooth muscle actin
FAP: Fibroblast activation protein
PDGFR: Platelet-derived growth factor receptor
TGF-β: Transforming growth factor-beta
PDAC: Pancreatic ductal adenocarcinoma
iCAFs: Inflammatory CAFs
myCAFs: Myofibroblastic CAFs
IL-1/6: Interleukin-1/6
JAK: Janus kinase
STAT: Signal transducer and activator of transcription
TGFBR: Transforming growth factor receptor
FSP-1: Fibroblast-specific protein-1
VEGF: Vascular endothelial growth factor
CCL2: Chemokine ligand 2
mAb: Monoclonal antibody
CAR: Chimeric antigen receptor
LUAD: Lung adenocarcinoma
EMT: Epithelial-to-mesenchymal transition
PDPN: Podoplanin
CAV1: Caveolin1
ER: Estrogen receptor
Saa3: Serum amyloid A3
scRNA-seq: Single-cell RNA sequencing
GPR77: G protein- coupled receptor 77
TNBC: Triple-negative breast cancer
CXCL12: CX-chemokine ligand 12
RGS5: Regulator of G-protein signaling 5
MHC: Major histocompatibility complex
apCAFs: Antigen presenting CAFs
ICC: Intrahepatic cholangiocarcinoma
vCAFs: Vascular CAFs
mCAFs: Matrix CAFs
MCAM: Melanoma cell adhesion molecule
POSTN: Periostin
FBLN1: Fibulin 1
KRT19: Keratin 19
SCRG-1: Stimulator of chondrogenesis 1
MMP2: Matrix metallopeptidase 2
DCN: Decorin
COL1A2: Collagen type I alpha 2 chain
TAGLN: Transgelin
THY1: Thy-1 cell surface antigen
HGSOC: High-grade serous ovarian cancer
